# Evaluation of Various Drying Methods for *Polygonatum cyrtonema* Hua: Effects on Drying Characteristics and Multidimensional Quality Assessment

**DOI:** 10.3390/foods15112035

**Published:** 2026-06-05

**Authors:** Liling Wang

**Affiliations:** Zhejiang Academy of Forestry, Hangzhou 310023, China; echo22239@163.com

**Keywords:** *Polygonatum cyrtonema* Hua, drying methods, multidimensional quality assessment, drying characteristics

## Abstract

The drying kinetics and quality attributes of *Polygonatum cyrtonema* Hua (PCH), including color, rehydration efficiency, microstructure, and oxidation resistance, were systematically evaluated under various drying methods: hot-air drying (H-D), infrared drying (IR-D), microwave drying (M-D), freeze-drying (F-D), and vacuum drying (V-D). The results indicate that the Midilli model provides the best fit for the experimental data. Among the five drying methods, hot air drying (H-D) is extensively utilized due to its well-established technology; however, its drying performance is relatively average. M-D and IR-D exhibit high drying rates attributed to their strong thermal permeability. Notably, M-D achieves the highest drying rate, with a drying time of only 1/83 that of F-D, yet it demonstrates a relatively low retention rate of total polysaccharides. Furthermore, while IR-D offers fast drying rates, low energy consumption, and favorable color preservation, it performs poorly in preserving the microstructure, active component content, and oxidation resistance of PCH. By contrast, F-D and V-D exhibit significant advantages in maintaining antioxidant activity, active ingredients, and flavor. Nevertheless, F-D requires an extended duration (1080 min), leading to high energy consumption, which restricts its large-scale industrial application. This comprehensive analysis revealed that V-D achieves an optimal balance between energy consumption and product quality, demonstrating substantial advantages and providing a critical reference for the industrial drying production of PCH.

## 1. Introduction

*Polygonatum cyrtonema* Hua (PCH) is a perennial herb of the family Liliaceae, whose dried rhizome is a typical edible traditional Chinese medicine with a long history documented in Shennong Materia Medica Classic. Modern studies have verified that PCH is rich in polysaccharides, steroid saponins, triterpenoids, and flavonoids, with various pharmacological activities, including regulating glucose and lipid metabolism [1], scavenging free radicals [2], enhancing immunity [3], alleviating acute lung injury [4], preventing alcoholic gastric injury [5], and delaying aging [6]. It is regarded as a valuable resource for developing antidiabetic, antitumor, and functional food products [7,8,9]. However, fresh PCH rhizomes contain 75–85% moisture and irritant components, such as saponins, that induce tongue numbness [9,10]. Direct consumption can cause toxic reactions, while improper storage easily leads to mold growth. Thus, drying is essential for dehydration, enzyme inactivation, and component transformation.

Drying is a critical step in the processing of Chinese medicinal materials. Drying quality directly affects active ingredient retention, appearance, safety, and thus medicinal efficacy and economic value [11,12]. Traditional drying methods, such as natural drying and hot-air drying are still widely used in major PCH-producing areas but have obvious drawbacks. Prolonged high temperature or UV irradiation causes oxidative degradation of heat-sensitive polysaccharides [13] and 30–50% flavonoid loss [14]. Intensified Maillard reactions may generate 5-hydroxymethylfurfural (5-HMF), a potential carcinogen that threatens product safety [15]. Moreover, differences in morphology, cell wall structure, and chemical composition among PCH sources (e.g., *Polygonatum filipes* and *Polygonatum sibiricum*) lead to unclear water migration and drying kinetics, resulting in poor adaptability of current drying processes and hindering standardized production of high-quality PCH slices. To address these issues, emerging drying technologies, including vacuum drying, infrared drying, microwave drying, and low-temperature adsorption drying, have attracted growing attention [16]. Freeze-drying removes water by sublimation under high vacuum, preserving nutrients, flavor, and microstructure while avoiding heat-induced degradation and discoloration [17,18]. Vacuum drying shortens the drying time by about 40% via pressure fluctuations and reduces browning under low-oxygen conditions [19,20,21]. Infrared-microwave drying achieves rapid dehydration through volumetric heating and directional radiation, but systematic studies on parameter optimization (e.g., power ratio and radiation distance) and saponin stability are still needed [22]. Drying optimization requires balancing component retention, safety, and efficiency. Low-temperature drying (<50 °C) protects polysaccharides but may prolong processing and increase microbial risk, while rapid dehydration can cause case hardening and trap internal moisture [19].

Current research on PCH mainly focuses on chemical composition and pharmacological activities, with few systematic comparisons of drying effects on morphology and quality, and no unified optimal drying protocol. Hot-air, vacuum, infrared, freeze-, and microwave drying are widely used in labs and industry, yet their drying kinetics and effects on bioactive components remain unclear. Studies on other herbs have shown that each method has unique advantages and limitations [12,14]. Therefore, a comprehensive comparative evaluation is essential to provide a scientific basis and theoretical support for optimizing PCH drying technology.

This study systematically investigated the effects of five industrial drying technologies—hot-air, vacuum, infrared, freeze-, and microwave drying—on the phenotype, composition, and antioxidant activity of PCH slices. Drying characteristics and kinetics were analyzed to evaluate the impacts on physical properties, including color, microstructure, water migration, and rehydration capacity. The advantages and disadvantages of each method were compared regarding active component content, antioxidant activity, and flavor retention. Using multi-scale and multi-objective indicators, this work comprehensively assessed drying performance, clarified the relationship between drying processes and quality attributes, and identified the optimal drying technology with high efficiency, good nutrient retention, and industrial applicability. The results provide a scientific basis and technical support for the development of PCH processing.

## 2. Materials and Methods

### 2.1. Reagents and Materials

PCH was collected from Liangxi Village, Zhuyang She Township, Lishui City, Zhejiang Province, China (118°56′47.92″ E, 28°03′5.99″ N, altitude 331.1 m). The samples were authenticated by Dr. Liu Bentong, a senior researcher at the Zhejiang Academy of Forestry Science. The samples were subsequently stored in cold storage at 4 °C until used. Prior to the formal experiments, the average moisture content of the fresh extract was determined to be 80.25% (wet basis, w.b.) and 432.17% (dry basis, d.b.) under room-temperature conditions. Subsequently, a multi-functional slicer (model DYQ-401B, manufactured by Rui’an Yongli Pharmaceutical Machinery Co., Ltd., Wenzhou, China) was employed to slice the PCH into irregular circular sheets with a thickness of 2 ± 0.2 mm for subsequent experimental procedures. The device’s cutting range was 0.3–3 mm, with an error tolerance of ±10%. At this stage, the average initial water content of the sample was 86.87% (w.b.) and 584.18% (d.b.).

High-performance liquid chromatography (HPLC) was employed to quantify the contents of sucrose, glucose, trehalose, fructose, and other components. The experimental reagents included chromatography-grade acetonitrile, phosphoric acid, and methanol. Additionally, Folin–Ciocalteu reagent, gallic acid, sodium nitrite, potassium persulfate, anhydrous sodium carbonate, rutin, sodium hydroxide, non-hydrated aluminum nitrate, ABTS [(2,2′-azino-bis(3-ethylbenzothiazoline-6-sulfonic acid)] radical cation, and DPPH (1,1-diphenyl-2-picrylhydrazyl) were utilized for the determination of total phenolic content, total flavonoid content, and antioxidant activity. Sulfuric acid and phenol were also employed for the quantification of total polysaccharide content.

### 2.2. Drying Experiments

In each replicate experiment, PCH slice samples weighing 100 ± 0.5 g were randomly selected and subjected to drying treatment. All samples were uniformly distributed on a single carrier tray fabricated from 304 stainless steel. The tray featured a straight mesh structure with a 30-mesh (0.6 mm) aperture and dimensions of 640 × 460 × 45 mm. This design was intended to minimize the contact area between the samples and the tray, thereby reducing heat transfer losses and enhancing drying efficiency. All drying experiments were performed in a temperature-controlled laboratory environment. In accordance with the provisions of the Chinese Pharmacopoeia (2020 Edition) and drying test experience, the drying process was terminated when the moisture content of the PCH samples reached the safety standard of 15% (wet basis, w.b.).

Microwave Drying (M-D): The sample was positioned in a microwave drying oven (model PM20A1, manufacturer: Midea Group Co., Ltd., Suzhou, China) for the drying process. The microwave operational frequency was set at 2450 MHz, with a rated input power of 3200 W. The ambient relative humidity was maintained at 4.00 ± 0.50%. During the experiment, the sample was removed at 1 min intervals, and its mass was measured to document the mass variation during each drying interval.

Infrared drying (IR-D): During the infrared drying process, the sample to be dried was positioned on the tray of an infrared drying oven (Model WS70-1, manufactured by Hangzhou Qiwei Instrument Co., Ltd., Hangzhou, China), maintaining a distance of 10 cm from the infrared heat source. The equipment was equipped with two infrared bulbs, each rated at 275 W, with an insulation resistance of 10 MΩ, total power output of 550 W, and ambient relative humidity controlled at 4.00 ± 0.50%. During the experiment, the sample mass was recorded every 5 min to monitor the mass changes during the drying process.

Hot-air drying (H-D): The sample to be dried was evenly distributed in a hot-air drying oven (Model GZX-9076 MBE, manufactured by Shanghai Boxun Industrial Co., Ltd., Medical Equipment Factory, Shanghai, China). The instrument parameters were set as follows: temperature at 50 °C, air speed at 0.5 m·s^−1^, relative humidity at 6.00 ± 0.50%, and working power at 1600 W. During the experiment, samples were removed and weighed every 30 min to document the mass changes during the drying process.

Vacuum drying (V-D): The sample to be dried was evenly placed in a vacuum oven (Model DZF-6020, manufactured by Shanghai Yiheng Scientific Instrument Co., Ltd., Shanghai, China), followed by activation of the vacuum pump. When the relative pressure reached −0.085 MPa, the vacuum process was halted. The drying temperature was set to 50 °C, and the instrument operated at a power of 1400 W. During the experiment, the sample mass was measured every 30 min to record the mass changes during the drying process.

Freeze-drying (F-D): The sample to be dried was first subjected to pre-freezing at −80 °C for a duration of 2 h. Subsequently, it was transferred to a vacuum freeze-drying oven (Model SCIENTZ-12, manufactured by Ningbo Xinzhi Biotechnology Co., Ltd., Ningbo, China) for the drying process. The vacuum pressure was maintained at 6.0 Pa, the cold trap temperature was set to −50 °C, and the instrument’s power rating was 1100 W. During the experiment, the sample mass was recorded at intervals of 120 min to monitor the quality changes throughout the drying process.

### 2.3. Drying Characteristics Analysis

#### 2.3.1. Analysis of Dry-Basis Moisture Content

The formula for calculating the dry-basis water content (*M*_d_) of PCH pieces is presented as follows:

(1) $$M_{d}=\frac{m_{1}-m_{2}}{m_{2}}\times 100\%$$

where *m*_1_ is the mass of the fresh sample (g); *m*_2_ is the dry basis mass of the sample (g).

#### 2.3.2. Moisture Ratio

The moisture content of the drying sample at time t can be transformed to be moisture ratio (*MR*) using the following equation (Equation (2)):

(2) $$MR=\frac{M_{d}-M_{g}}{M_{0}-M_{g}}$$

At the end of drying, the dry-basis water content of the sample is much smaller than the initial dry-basis water content of the sample. Therefore, the calculation formula can be simplified as follows:

(3) $$MR=\frac{M_{d}}{M_{0}}$$

where *M*_d_ represents the dry-basis water content of PCH at a specific time (g·g^−1^); *M*_0_ and *M*_g_ are the initial moisture content (g⋅g^−1^ d.b.) and equilibrium moisture content (g⋅g^−1^ d.b.), respectively.

#### 2.3.3. Drying Rate

The drying rate (DR) of PCH pieces is an essential index for determining the drying properties and can be computed using Equation (4):

(4) $$DR=\frac{M_{t}-M_{t+\Delta t}}{\Delta t}$$

where *DR* is the material drying rate (g⋅g^−1^⋅min^−1^); *M*_t+Δt_ represents the dry-basis water content of PCH pieces at time *t* + Δ*t* (g⋅g^−1^ d.b.); and *M_t_* refers to the dry-basis water content of PCH pieces at time *t* (g⋅g^−1^ d.b.).

#### 2.3.4. Mathematical Modeling of Drying Curves

According to the drying characteristics of PCH medicinal materials, six commonly used mathematical models were selected to fit and validate the experimental data obtained from various drying methods (Table 1). The tested samples were irregular circular sheets with a cross-sectional geometry identical to that of natural polygonatum rhizome. For the diffusion calculation, ideal diffusion boundary conditions were adopted: moisture migration occurred uniformly inside the sample, and the moisture content at the material surface rapidly reached equilibrium with the ambient drying atmosphere. No internal mass loss and structural damage were assumed during the diffusion process. Data processing was performed using ORIGIN 2019b data analysis software, and the mathematical model equations were fitted to the experimental data via nonlinear regression analysis. The fitting quality of the mathematical models was assessed using the coefficient of determination (*R*^2^), root mean square error (RMSE), and sum of squared deviations (χ^2^). Specifically, a higher *R*^2^ value and lower RMSE and χ^2^ values indicate superior fitting performance [23]. The detailed calculation formulas are presented in Equations (5)–(7):

(5) $$R^{2}=1-\frac{\sum_{i=1}^{n}{\left({MR}_{I,pre}-{MR}_{i,exp}\right)}^{2}}{\sum_{i=1}^{n}{\left({MR}_{I,exp}-{MR}_{{i,pre}_{mean}}\right)}^{2}}$$

(6) $$\chi^{2}=\frac{\sum_{i=1}^{N}{\left({MR}_{exp,i}-{MR}_{pre,i}\right)}^{2}}{N-z}$$

(7) $$RMSE=\sqrt{\frac{\sum_{i=1}^{n}{\left({MR}_{exp,i}-{MR}_{i,exp}\right)}^{2}}{N}}$$

where *MR_i_*_,*exp*_ is the moisture ratio measured experimentally at any given drying time; *MR_i_*_,*pre*_ is the predicted moisture ratio of PCH under the same drying conditions; *N* represents the total number of experimental data points; and *z* denotes the number of constants in the model equation.

### 2.4. Scanning Electron Microscopy (SEM) Observation and Rehydration Capacity Determination

#### 2.4.1. SEM 

The microstructure of each treated sample was observed using scanning electron microscopy (SEM) (Thermo Fisher Apreo 2S, Waltham, MA, USA).

#### 2.4.2. Rehydration Capacity Determination

A 10.0 ± 0.1 g quantity of the PCH sample was added to distilled water at a constant temperature of 25 ± 0.1 °C. The sample was removed at 30 min intervals, the surface moisture was carefully blotted using filter paper, and the weight was precisely recorded. This procedure was continued until the sample weight reached equilibrium. Thereafter, the rehydration rate of the PCH samples was calculated according to Equation (8):

(8) $$RR=\frac{m_{3}}{m_{4}}$$

where *RR* denotes the rehydration ratio of PCH, m_3_ represents the mass of the PCH sample after rehydration (g), and m_4_ represents the mass of the PCH sample before rehydration (g).

The rehydrated samples were set with formalin–acetic acid–ethanol solution for 48 h, followed by dehydration, clarification, paraffin embedding, sectioning into 2 μm slices using a microtome, mounting on glass slides, and drying. The slices were stained with toluidine blue for observation of the rehydrated PCH sample structures under a vertical optical microscope (NIKON ECLIPSE E100, Tokyo, Japan). Images were captured at 40× magnification using an imaging system (NIKON DS-U3, Tokyo, Japan).

### 2.5. Color Analysis

The L*, a*, and b* color values of the samples were measured using a CM-5 colorimeter (Konica Minolta, Tokyo, Japan), with each group randomly measured three times. Here, L* represents the lightness index, a* indicates the red–green value, and b* denotes the yellow–blue value. The total color change (ΔE) for each sample was calculated using Equation (9):

(9) $$∆E=\sqrt{{\left(L-L_{0}\right)}^{2}+{\left(a-a_{0}\right)}^{2}+{\left(b-b_{0}\right)}^{2}}$$

where ΔE represents the total color difference of the sample; and L_0_, a_0_, and b_0_ denote the brightness, red–green, and yellow–blue values of fresh PCH pieces, respectively. L, a, and b represent the corresponding values for the dried PCH sheet. The Browning index (BI) was calculated using Equations (10) and (11):

(10) $$BI=\frac{100(x-0.31)}{0.172}$$

(11) $$X=\frac{a+1.75L}{5.645L+a-3.012b}$$

### 2.6. Physicochemical Analysis

#### 2.6.1. Sample Preparation

The PCH samples, which were subjected to various drying methods, were ground using a mill and subsequently sieved for further use. A 1.0 g quantity of the PCH powder was accurately weighed and transferred into a 50 mL conical flask. Next, 10 mL of 80% ethanol was added and mixed thoroughly. Ultrasound-assisted extraction was performed at an extraction temperature of 30 °C for 25 min. After cooling to room temperature, the mixture was filtered and the filtrate was collected and stored in a refrigerator at 4 °C for subsequent analysis.

#### 2.6.2. Total Flavonoids and Total Phenols

The determination of total flavonoid content (TFC) was performed with reference to the corresponding method [24], with appropriate adjustments made. A 1 mL aliquot of the sample solution was accurately measured and transferred into a test tube. Subsequently, 0.1 mL of a 5% sodium nitrite solution (*w*/*v*) was added, and the mixture was incubated for 5 min. Then, 0.1 mL of a 10% aluminum nitrate solution (*w*/*v*) was introduced, followed by an additional incubation period of 6 min. Finally, 0.8 mL of a 1.0 mol·L^−1^ NaOH solution was added, and the resulting mixture was allowed to react for 10 min. The absorbance was measured at a wavelength of 510 nm using a microplate reader (Thermo Fisher Scientific, Waltham, USA), with the zero tube serving as the blank control. Rutin was used as the standard reference, and the linear regression equation obtained was Y = 0.0031X + 0.0388 (*R*^2^ = 0.9997), where Y represents the absorbance value and X denotes the concentration of rutin (μg·mL^−1^). The results are expressed as the equivalent amount of rutin (mg/g) per gram of dry weight.

The determination of total phenolic content (TPC) was conducted with slight modifications to the corresponding method [25]. A 0.04 mL aliquot of the sample solution was taken and diluted to 1 mL with water. Subsequently, 0.1 mL of Folin reagent was added, and the mixture was thoroughly vortexed and incubated for 8 min. Then, 0.3 mL of a 20% (*w*/*v*) sodium carbonate aqueous solution was added, followed by incubation in the dark at room temperature for 2 h. The absorbance was measured at a wavelength of 760 nm using a microplate reader (Thermo Fisher Scientific, Waltham, USA), with the zero tube serving as the blank control. Gallic acid (GAE) was used as the standard reference, and the linear regression equation obtained was Y = 0.0878X + 0.0993 (*R*^2^ = 0.9991), where Y represents the absorbance value and X denotes the concentration of gallic acid (μg·mL^−1^). The results are expressed as milligrams of GAE equivalents per gram of dry weight (mg GAE·g^−1^ dw).

#### 2.6.3. Antioxidant Activity

The antioxidant activity of PCH was assessed via DPPH and ABTS radical scavenging and ferric reducing antioxidant power (FRAP) methods according to Bhat et al. [26], with some modifications.

##### DPPH Free Radical Scavenging Activity

A 0.1 mL aliquot of the sample solution was taken, and 3.9 mL of DPPH solution (0.101 mmol·L^−1^) was added. After reacting at room temperature for 1 h, the absorbance was measured at 517 nm. A blank control consisting of 70% methanol was used. The standard curve was constructed with vitamin C (VC) concentration as the *x*-axis and absorbance as the *y*-axis. The linear regression equation obtained was Y = −0.0351X + 0.8359 (*R*^2^ = 0.9992), where Y represents the absorbance value and X represents the VC concentration (μg·mL^−1^). The results are expressed as the equivalent amount of VC per gram of dry weight (mg VC·g^−1^ dw), and all experiments were conducted in triplicate.

##### ABTS Free Radical Scavenging Assay

A K_2_S_2_O_8_ solution (140 mmol·L^−1^) and an ABTS stock solution (7 mmol·L^−1^) were prepared. Subsequently, 176 μL of the K_2_S_2_O_8_ solution was mixed with 10 mL of the ABTS stock solution and incubated in the dark at room temperature for 12–16 h. Following the reaction, the resultant ABTS solution was diluted with ethanol to achieve an absorbance value of 0.700 ± 0.02 at 734 nm. For the assay, 0.1 mL of the sample solution was added to 3.9 mL of the ABTS˙+ working solution, mixed thoroughly, and reacted at room temperature for 6 min. The absorbance of the resulting solution was measured at 734 nm, using 70% methanol as the blank control. A standard curve was constructed by plotting the concentration of VC on the *x*-axis and the absorbance values on the *y*-axis. The linear regression equation obtained was Y = −0.0042X + 0.6782 (*R*^2^ = 0.9992), where Y represents the absorbance value and X represents the VC concentration (μg·mL^−1^). The results are expressed as the equivalent amount of VC per gram of dry weight (mg VC·g^−1^ dw), and all experiments were performed in triplicate.

##### Ferric Ion Reducing Antioxidant Power (FRAP) Assay

A 0.1 mol·L^−1^ acetic acid buffer (pH 3.6), a 10 mmol·L^−1^ TPTZ solution (dissolved in 40 mmol·L^−1^ hydrochloric acid), and a 20 mmol·L^−1^ ferric chloride solution were prepared. The FRAP working solution was subsequently formulated by mixing these reagents at a ratio of 10:1:1 (*v*/*v*/*v*). To conduct the assay, 3.9 mL of the working solution was added to 0.1 mL of the sample solution, followed by thorough mixing. The mixture was incubated in a water bath at 37 °C for 10 min. A blank control was established using 70% methanol. Absorbance was measured at 593 nm. A standard curve was constructed with ferrous sulfate concentration (mmol·L^−1^) as the *x*-axis and absorbance as the *y*-axis. All experiments were performed in triplicate to ensure reproducibility. The resulting linear regression equation was Y = 3.4289X + 0.135 (*R*^2^ = 1), where Y represents absorbance and X denotes the concentration of ferrous sulfate (mmol·L^−1^). The results are expressed as the equivalent amount of ferrous sulfate per gram of dry weight (mmol Fe(II)/g).

#### 2.6.4. Carbohydrates

##### Total Polysaccharides

After freeze-drying, approximately 0.1 g of the sample was accurately weighed. A 3 mL volume of distilled water was added, and the mixture was thoroughly homogenized. Ultrasonic extraction was subsequently performed at 80 °C for 3 h. The extract was cooled and then centrifuged at 8000 rpm for 10 min. A volume of 0.3 mL of the supernatant was carefully aspirated, followed by the addition of 1.2 mL of anhydrous ethanol. The solution was mixed and left to precipitate at 4 °C for 12 h. Afterward, the mixture was centrifuged again at 8000 rpm for 10 min, and the supernatant was discarded. Finally, 0.5 mL of distilled water was added to the precipitate to dissolve it, yielding the solution to be measured. A 25 μL volume of the test solution was taken, and 50 μL of a 5% phenol solution was added. The mixture was thoroughly combined, followed by the slow addition of 175 μL of concentrated sulfuric acid. The resulting solution was mixed well and subjected to a boiling water bath for 10 min. After removal from the water bath, the solution was cooled and allowed to stand at room temperature for 30 min. The absorbance value was then determined at a wavelength of 490 nm. Using glucose as the reference standard, the linear regression equation obtained was Y = 1.115x + 0.073, where Y represents the absorbance and x denotes the glucose concentration (mg·mL^−1^). The polysaccharide content in the sample was calculated using the equation: Total Polysaccharide (mg·g^−1^) = C/(V_A_ × V_E_× V_C_/M × F × 0.9), where C represents the concentration of glucose calculated from the standard curve (mg·mL^−1^), V_E_ represents the volume of the extraction solution (3 mL), V_A_ represents the volume of alcohol precipitation (0.3 mL), V_C_ represents the constant volume used (0.5 mL), M represents the mass of the sample (g), F represents the dilution factor, and 0.9 represents the correction factor for converting glucose to dextran.

##### Soluble Sugars

Approximately 0.25 g of PCH powder was accurately weighed and transferred into a stoppered conical flask, and exactly 50 mL of a 25% ethanol solution was added. The flask was sealed, weighed again, and subjected to ultrasonic extraction for 40 min. After cooling, the flask was reweighed, and the 25% ethanol solution was used to compensate for any loss. The solution was passed through a 0.22 μm filter membrane to obtain the sample. The chromatographic conditions for the determination of fructose, sucrose, glucose, and trehalose were as follows: the chromatographic column used was an Asahipak NH2P-50 4E (Shodex, Tokyo, Japan) (4.6 mm × 150 mm, 5 μm); the mobile phase composition was acetonitrile–water (75:25) over 0–20 min; the drift tube temperature was maintained at 90 °C, with a gas flow rate of 2.2 mL·min^−1^; the column temperature was set at 30 °C; the injection volume was 5 μL; and the flow rate was adjusted to 1.0 mL·min^−1^.

#### 2.6.5. Flavor Compounds

The SPME fiber (SPME handle, Supelco, extraction needle coated with 65 μm PDMS/DVB) was conditioned at 250 °C for 3 min. A 2.0 g sample was accurately weighed and transferred into a 15 mL headspace vial, ensuring that at least one-third of the vial remained empty for headspace generation. The conditioned SPME fiber was inserted into the headspace of the vial, and adsorption was performed at 40 °C for 50 min. Subsequently, the fiber was thermally desorbed in the GC inlet for 2 min. The gas chromatographic conditions were as follows: DB-5ms column (30 m × 0.25 mm, 0.25 μm film thickness); helium carrier gas; inlet temperature of 250 °C; constant flow rate of 1 mL·min^−1^; initial oven temperature of 50 °C held for 1 min, then increased to 260 °C at a rate of 5 °C·min^−1^, followed by a 3 min hold at 260 °C. The mass spectrometry conditions were as follows: interface temperature of 280 °C; ion source temperature of 230 °C; scanning mass range of 15–550 amu; and qualitative analysis using the NIST20 library.

The flavor characteristics were obtained through a review of relevant scientific literature and authoritative databases (https://www.femaflavor.org/; https://www.metaboanalyst.ca/; https://www.flavornet.org/flavornet.html; https://www.odour.org.uk/odour/index.html?i=1) (accessed on 8 May 2025).

### 2.7. Statistical Analysis

The experimental data were statistically processed using SPSS 16.0 software, with the results expressed as the mean ± standard deviation (SD, *n* = 3); *p* < 0.05 was regarded as the criterion for determining the significance of difference. The mathematical model equations for drying dynamics were fitted based on the experimental data presented in Table 1, and the corresponding model parameters were derived using Origin 2012 software (Origin Lab, Northampton, MA, USA).

## 3. Results and Discussion

### 3.1. Drying Features

**Figure 1:**
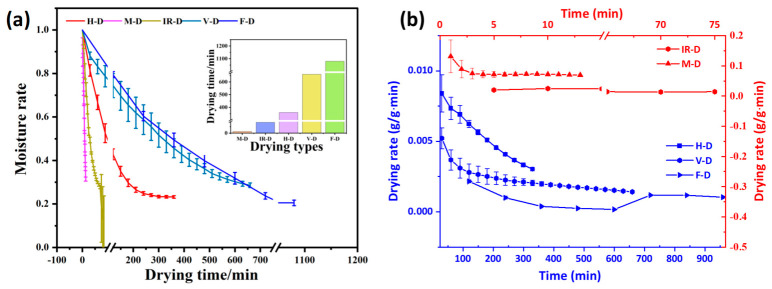
(**a**) Moisture ratio vs. drying time curves of PCH and drying time comparison for 5 drying methods; (**b**) Drying ratio vs. drying time curves of PCH.

As illustrated in Figure 1a, the drying durations of the five methods exhibited substantial variation, ranging from 13 to 1080 min. Despite this disparity, the drying curve trends were largely consistent, exhibiting an exponential decline. Microwave drying (M-D) exhibited superior performance, characterized the shortest drying time of 13 min and the steepest curve slope. This can be attributed to the distinctive dual-action mechanism of microwaves: (1) high-frequency electromagnetic waves penetrate the material, generating molecular frictional heat; and (2) the pressure gradient created by the vacuum environment accelerates water migration [27]. By contrast, infrared drying (IR-D) is constrained by its surface heating characteristics, resulting in a significantly longer drying time of 85 min. Traditional hot-air drying (H-D) required 360 min due to the limitations of convective heat transfer efficiency, while simple vacuum drying (V-D) extended the drying time to 660 min to maintain a low-pressure steady state [28,29]. Freeze-drying (F-D), as a specialized phase transition process involving pre-freezing, sublimation, and desorption stages, demanded the longest drying time of 1080 min, which is directly associated with the kinetic characteristics of ice crystal sublimation [30].

Analysis of the drying rate revealed that all PCH samples experienced an initial significant decline, followed by a progressive stabilization of the drying process (Figure 1b). This phenomenon can be attributed to the high content of free water within the cells during the early stages of drying, which facilitates rapid water loss upon exposure to the heat source or during the freezing process. Consequently, as thermal equilibrium was progressively achieved both internally and externally within the material, the drying rate reached a stable state [31]. Under identical dry-basis water content conditions, the drying rates were ranked as follows: M-D > IR-D > H-D > V-D > F-D.

The effective water diffusion coefficient (Deff) serves as a critical parameter for characterizing intrinsic mass transfer performance and reflecting water diffusion behavior. A higher Deff value indicates superior drying potential [32,33]. As presented in Table 2, the determination coefficient *R*^2^ of lnMR vs. Time (min) linear fitting for the five drying methods ranged from 0.939 to 0.996, demonstrating an excellent fitting quality. The Deff values ranged from 4.836 × 10^−11^ to 1.741 × 10^−9^ m^2^·s^−1^, falling within the typical diffusion coefficient range of 10^−11^ to 10^−9^ m^2^·s^−1^ for most agricultural products [34]. Notably, M-D exhibited the highest Deff value (1.741 × 10^−9^ m^2^·s^−1^), indicating the most efficient moisture transfer. This phenomenon is caused by the intense fluctuation of polar molecules, such as water, in a microwave electric field, which generates a significant substantial heat. Consequently, a significant pressure gradient is established between the interior and the surface of the product, facilitating rapid water migration and evaporation [35]. By contrast, F-D and V-D exhibited the lowest Deff values (7.255 × 10^−11^ and 4.836 × 10^−11^ m^2^·s^−1^, respectively), which were of the same order of magnitude. These low values can be attributed to the vacuum environment, which reduces the kinetic energy of water molecules, decreases the rate of evaporation, and diminishes their diffusion capacity [36]. The Deff values for IR-D and H-D were 5.320 × 10^−10^ and 2.297 × 10^−10^ m^2^·s^−1^, respectively. Hot-air drying heats materials from the surface inward, opposing the direction of water diffusion, whereas infrared drying heats both the interior and exterior simultaneously, aligning with the direction of water diffusion. Therefore, the water diffusion coefficient of hot-air drying was smaller than that of infrared drying. In summary, in the drying process, the moisture mass transfer efficiencies were ranked as follows: M-D > IR-D > H-D > F-D > V-D.

Drying, a nonlinear process characterized by coupled heat and mass transfer, exhibits kinetic behavior influenced by both material properties and process parameters [37]. This study systematically evaluated the characterization capabilities of six classical drying models for the dehydration behavior of PCH sections, revealing notable differences in their fitting performance (Table 3). Model optimization was guided by a three-dimensional evaluation system comprising a higher coefficient of determination (R^2^) and lower root mean square error (RMSE) and chi-square (χ^2^) values. The verification results indicated that the Midilli model exhibited superior applicability in describing the dehydration kinetics of PCH sections. The advantages of this model are substantiated by the following features: first, the Midilli model demonstrated optimal overall performance, with its R^2^ values (0.97894–0.99951) ranking highest among all models; and second, its error indicators were the lowest, with χ^2^ (0.001187–2.17 × 10^−5^) and RMSE (0.004663–0.034456) achieving the lowest levels in their respective categories. Experimental validation confirmed that the model’s prediction error for rapid drying processes, including H-D and IR-D, remained within 0.1%, and it was capable of accurately capturing the dynamics of the sublimation front during complex phase transition processes such as F-D. Based on these findings, it is recommended to integrate the Midilli model into the PCH drying process optimization system, as its formulation in terms of differential equations is particularly well-suited for the development of real-time control systems.

### 3.2. SEM Observation

**Figure 2:**
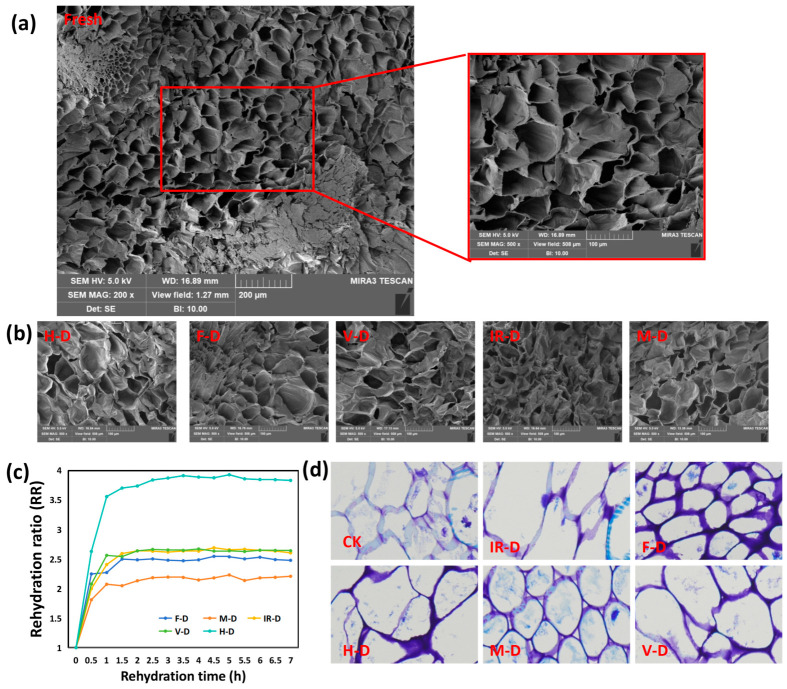
(**a**) The microstructure of fresh PCH magnified at ×200 and ×500); (**b**) PCH samples obtained from five different drying methods (magnified at ×500) examined via SEM; (**c**) The rehydration curves of PCH obtained from the five drying methods; (**d**) The microstructure of fresh PCH and rehydrated PCH samples obtained from five different drying methods (magnified at ×40).

SEM characterization demonstrated that the fresh PCH samples, without undergoing drying treatment, exhibited typical plant tissue structural characteristics. Their vascular bundles were arranged in a complete annular configuration, the three-dimensional network of the cell wall remained intact, and starch particles were uniformly distributed within the porous matrix (Figure 2a). The preservation of biological structural integrity can be attributed to the lubricating effect of interstitial water and the stabilizing role of the hydrogen bond network within the cell wall. Both hot-air drying and microwave drying induced the formation of a liquid water/vapor biphase interface at the sample surface, thereby generating surface tension [38]. This surface tension promoted the migration and aggregation of starch particles, as well as the folding and collapse of the cell wall. The former process led to the development of radial cracks, while the latter resulted in reticular fracture zones, causing significant deformation, loosening, and collapse of the tissue structure. Infrared radiation drying (IR-D) triggered instantaneous internal vaporization due to the penetration of the infrared band through the epidermis. The resulting vapor pressure exceeded the tensile strength of the cell wall, embedding starch particles into the rigid matrix and forming distinct morphological features, such as convolution, wrinkling, and cracking [39]. F-D effectively mitigated severe structural deformation through the sublimation of water under high-vacuum conditions [40]. However, it also led to loosely structured PCH tissues characterized by a lighter texture, fragile surface structures, and a higher tendency toward fragmentation. By contrast, vacuum drying (V-D) preserved a relatively smooth and uniform overall structure, with minimal curling or wrinkling observed. This preservation of the original structure can be ascribed to the effect of a balanced low-pressure environment, which not only helps maintain the balance inside and outside the sample but also promotes the loss of moisture while maintaining structural integrity. The findings of this study closely align with those observed during the drying process of other traditional Chinese medicines [14].

### 3.3. Rehydration Capacity

The rehydration curve results (Figure 2c) indicated that the H-D method exhibited superior rehydration performance compared to other methods. The rehydration capabilities of IR-D and V-D were comparable, while F-D demonstrated slightly inferior performance, and M-D showed relatively poor rehydration efficiency. This phenomenon is closely related to the significant shrinkage and structural collapse observed in the SEM images after drying treatment, further verifying that the damage caused by different drying techniques to the cell structure was irreversible to a certain extent. The microstructural analysis of the rehydrated samples (Figure 2d) indicated that the rehydrated structures of samples processed using the IR-D, V-D, and M-D methods were highly comparable to those of fresh PCH samples, exhibiting well-preserved cellular structures, sufficient moisture content, and clearly defined cell boundaries. These findings suggest that these methods effectively mitigate irreversible damage to cellular structures throughout the drying procedure. On the other hand, the PCH samples treated using the F-D and H-D approaches exhibited significant cell contraction phenomena, indicating that the dehydration process caused substantial damage to the internal architecture of the cellular tissues. Notably, in F-D samples, the destruction of cellular structures during freeze-drying led to cytoplasmic leakage and the accumulation of toluidine blue dye within the intercellular spaces.

### 3.4. Color Feature

**Figure 3:**
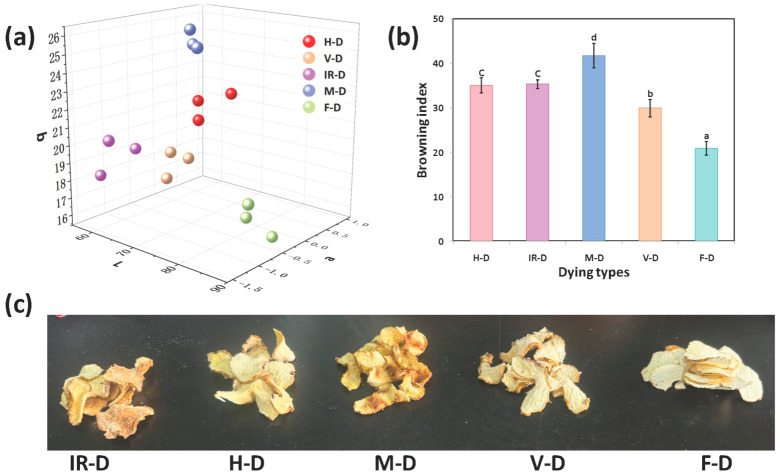
(**a**) Three-dimensional projection of L, a, and b values; (**b**) Comparison of browning index of different drying methods. Different lowercase letters indicate significant differences at *p* < 0.05 as determined by the LSD test; (**c**) Samples of PCH dried by five drying methods.

Chroma constitutes the primary factor for evaluating the appearance of a product. As shown in Figure 3a, in the three-dimensional color space defined by luminance (L*), red–green value (a*), and yellow–blue value (b*), the PCH samples prepared by five different drying methods showed significant differences and could be clearly distinguished. This indicated notable variations in their color properties. Among these, the freeze-dried (F-D) sample exhibited relatively higher brightness with lower a* and b* values, which can be attributed to the minimal thermal exposure during the drying process. By contrast, microwave-dried (M-D) samples displayed the highest b* value and the lowest L* value, resulting in an overall darker color. This phenomenon is attributed to the strong frictional heating effect of microwave radiation on water molecules, which intensifies the Maillard reaction and caramelization processes in PCH, leading to sample browning. By comparison, the color characteristics of PCH samples dried using H-D and IR-D were closely aligned, exhibiting brighter hues than those obtained by microwave drying. The color of V-D samples fell between that of freeze-dried and H-D samples. The ΔE values of the samples prepared by the five drying methods relative to fresh PCH were ranked as follows: IR-D (6.62 ± 0.89) < V-D (10.50 ± 2.85) < H-D (13.93 ± 2.68) < M-D (17.03 ± 2.47) < F-D (23.60 ± 1.56). These findings suggested that the color characteristics of samples processed using the IR-D method exhibited the closest resemblance to those of fresh PCH, while statistically significant differences were observed among all experimental groups.

Thermal effects and oxidation are considered key factors that promote the browning reaction [41]. Furthermore, prolonged drying duration increases the material’s exposure to oxygen, thereby enhancing the extent of browning. Significant heat transfer occurs during microwave and infrared drying processes, while the H-D method involves a longer heating period, leading to greater cumulative thermal exposure. Consequently, the BI values associated with these three drying methods were markedly higher than those of the other two methods (Figure 3b,c). By contrast, freeze-drying effectively minimizes both thermal effects and oxygen exposure, thereby suppressing the browning reaction to the greatest extent, resulting in the lowest browning index among all methods evaluated.

In summary, the temperature and drying duration of the drying process are the primary factors influencing the color change of materials [42]. Furthermore, different principles of action mechanisms also influence the outcomes. By maintaining relatively moderate temperatures and controlled drying durations, infrared drying preserved a color profile that more closely resembled that of fresh PCH.

### 3.5. TPC, TFC, and Antioxidant Activity of PCH

**Figure 4:**
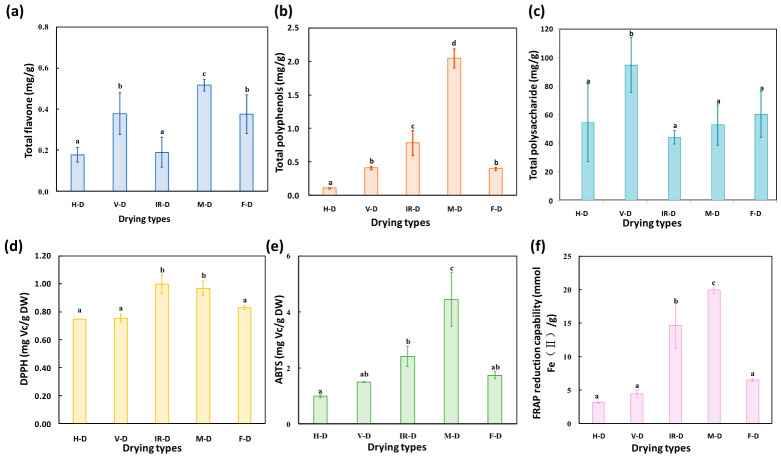
The total phenolic content (**a**), total flavonoid content (**b**) and total polysaccharide content (**c**) of PCH extracts. Antioxidant capacity of PCH extracts prepared with different drying methods: the scavenging rate of DPPH⋅ (**d**), the scavenging rate of ABTS+• (**e**), FRAP reduction capability (**f**). Different lowercase letters indicate significant differences at *p* < 0.05 as determined by the LSD test.

Numerous studies have confirmed that PCH is rich in bioactive substances, such as phenolic compounds, polysaccharides, and flavonoid components, which are directly associated with its pharmacological activities [43,44,45,46]. Comparative experiments (Figure 4a,b) revealed significant differences in the retention of active ingredients among the various drying methods. M-D, characterized by its short-time high-temperature feature, demonstrated superior performance compared to other methods in preserving total TPC and TFC. The reduced processing time effectively inhibited the oxidative degradation of heat-sensitive components. H-D, however, has limitations due to the continuous high-temperature environment, which intensifies the polymerization reaction of phenolic substances and increases the chain breakage degradation of glycosidic bonds in flavonoids [20]. Vacuum drying exhibits a unique advantage, as its low-oxygen environment reduces the breakage of polysaccharide molecular chains, while higher relative humidity enhances the retention rate of polysaccharides. Experimental data indicated a significant correlation (*p* < 0.05) between drying process parameters and the stability of active ingredients, providing a critical foundation for optimizing the processing technology of functional foods.

In summary, the effects of various drying methods on different components of PCH pieces were significantly distinct. M-D effectively suppressed the oxidation and degradation of total phenols and flavonoids, while V-D efficiently prevented the decomposition and degradation of total polysaccharides. If the objective is to preserve total polysaccharides in PCH, V-D should be chosen. Conversely, if the focus is on retaining active small molecules such as polyphenols and flavonoids, M-D is recommended to better maintain chemical stability and biological activity.

The assessment of free radical scavenging capacity, including DPPH•, ABTS+• scavenging tests, and FRAP determination, serves as a critical indicator for evaluating the antioxidant properties of functional foods [47]. The experimental results demonstrated that samples treated with different drying methods exhibited significantly distinct antioxidant properties. Notably, the highest DPPH clearance rate was observed in infrared-dried and microwave-dried samples. In both the ABTS+• clearance test and FRAP determination, M-D samples demonstrated superior performance. Conversely, all three indicators for H-D samples were at the lowest levels. This phenomenon can be attributed to the fact that instantaneous drying processes (e.g., M-D and IR-D) preserve the ability to scavenge free radicals by inhibiting the polymerization of phenolic compounds. By contrast, continuous thermal exposure induced irreversible structural alterations in the antioxidant components of H-D samples. Additionally, IR-D samples exhibited an antioxidant capacity comparable to that of M-D samples. These results were in close alignment with the measured values of TPC and TFC, suggesting that these two drying methods were markedly superior to the other three methods in maintaining higher antioxidant potential and quality.

### 3.6. Soluble Sugar of PCH

The soluble sugars (glucose, sucrose, trehalose, fructose, mannose, D-galactose, D-sorbitol, maltose, D-xylose, and L-rhamnose) in the samples obtained by various drying methods were analyzed. Four types of soluble sugars were detected: glucose, sucrose, trehalose, and fructose. Among these, fructose exhibited the highest content, followed by sucrose, while glucose and trehalose showed relatively low concentrations. The total amounts of the four soluble sugars obtained by the five drying methods were ranked as follows: V-D (102.96 ± 21.33 mg/g) > F-D, (101.45 ± 11.19 mg/g) > IR-D (75.47 ± 16.73 mg/g) > H-D (66.52 ± 9.23 mg/g) > M-D (42.04 ± 8.04 mg/g). F-D and V-D demonstrated significant advantages in retaining soluble sugar content. This is primarily attributed to the lower temperatures during F-D, which effectively suppress the activity of enzymes responsible for soluble sugar decomposition. Additionally, the V-D process establishes a negative pressure environment that not only prevents the degradation of sugar compounds but also minimizes oxygen involvement in reactions. This effectively suppresses the Maillard reaction between sucrose and amino acids, thereby better preserving the soluble sugar content. This finding is in line with the changes in soluble sugar content caused by drying methods in other species, such as white *Hypsizygus marmoreus* [48]. Detailed regression equations and their linear ranges are provided in Supplementary Material Table S1.

**Figure 5:**
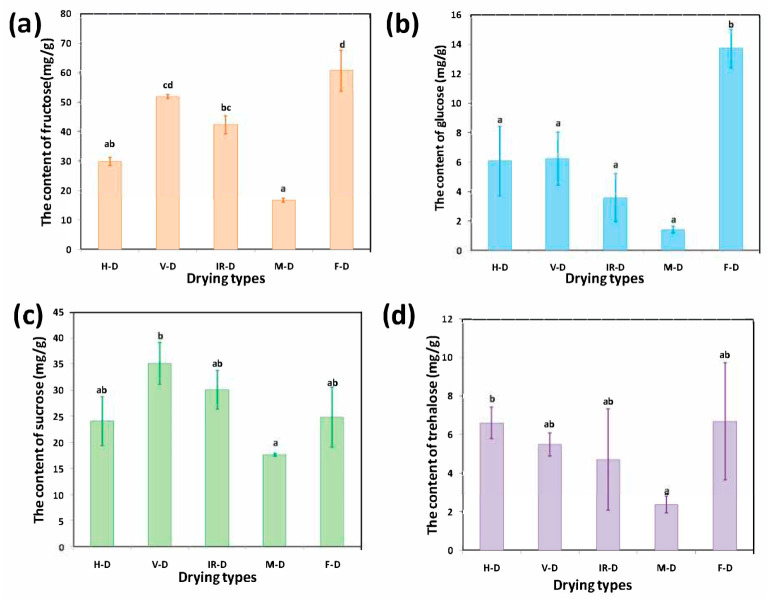
The content of soluble sugars: fructose (**a**), glucose (**b**), sucrose (**c**), trehalose (**d**). Different lowercase letters indicate significant differences at *p* < 0.05 as determined by the Tukey test.

In this tuber plant, fructose constitutes the primary product of sucrose hydrolysis, which is the main product of photosynthesis. The high concentration of fructose was consistent with the principles that govern carbohydrate storage in plants. Fructose exhibits excellent thermal stability (with a decomposition temperature of approximately 105 °C) and remains stable under various drying methods, such as H-D (typically at 60–80 °C), IR-D (with controllable temperatures), and others. Upon analysis of the content of each component, fructose levels obtained through V-D and F-D were found to be significantly higher than those achieved with the other four drying methods (Figure 5a). Conversely, M-D resulted in lower concentrations of all four types of soluble sugars, particularly glucose. This phenomenon may be attributed to the elevated temperatures during M-D (up to 250 °C), which can induce degradation and transformation of soluble sugars [49]. Trehalose, acting as a non-permeable protective agent, showed no significant variation in its content across the H-D, V-D, IR-D, and F-D groups (Figure 5d). However, M-D samples exhibited the lowest trehalose content, potentially due to its degradation and transformation caused by microwave drying and high temperatures. Additionally, glucose is highly reactive in Maillard reactions during high-temperature drying processes, such as H-D. However, its content remained consistently low across all groups. This is likely attributed to the high glucokinase activity inherent to PCH, which continuously catalyzes the conversion of glucose into fructose.

### 3.7. Flavor Components by GC-MS

**Figure 6:**
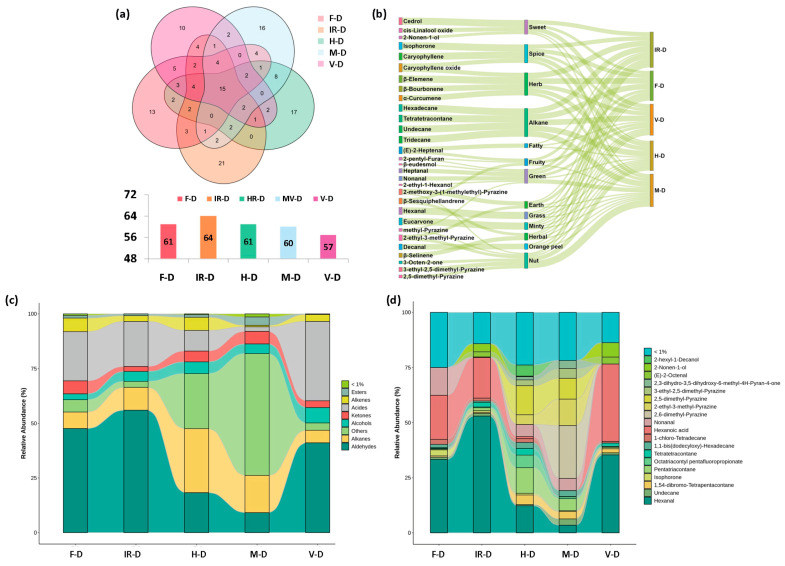
The flavor compounds Venn diagram of different drying methods for PCH (**a**) (The number represents the quantity of the compound.), the “Component–Flavor–Drying Method” correlation diagram of different drying methods for PCH (**b**), stacked column chart of flavor types (**c**) and components (**d**).

A total of 101 flavor compounds were detected in this study (Figure 6a), with their distribution quantified in the following order: IR-D (64) > F-D (61) = H-D (61) > M-D (60) > V-D (57). Among these, 15 aroma molecules, such as geraniol, n-chitosan, terpene brain acid, and isophorone, remained detectable across all tested drying approaches, indicating their thermal stability. Advanced profiling identified 21 method-specific volatile markers in IR-D samples, notably β-eudesmol and 5-oxo-isophorone, suggesting unique thermal reaction pathways. V-D samples exhibited distinct flavor characteristics, such as 8-oxo-isophorone and 2-amylfuran. Additionally, M-D, H-D, and F-D samples contained 16, 17, and 13 unique flavor components, respectively. Notably, other flavor compounds demonstrated partial overlap between different drying methods, suggesting potential interactions among the flavor profiles of each method.

These flavor components delineated 13 defining aroma attributes in post-dried PCH, encompassing sweet, spicy, herbal, alkane, fatty, fruity, green, earthy, grassy, minty, herbal, orange peel, and nutty (Figure 6b). Chromatographic peak areas, serving as quantitative proxies for volatile abundance, facilitated the development of a triadic analytical matrix that integrated chemical profiles, sensory attributes, and drying protocols (Figure 6c). The analysis revealed that the five drying methods exhibited similar sensory profiles in terms of alkane, grassy, minty, and herbal flavors, presenting soft herbaceous, minty, and alkane notes. By contrast, M-D samples demonstrated a more pronounced nutty flavor, while F-D samples exhibited a more significant herbal flavor, primarily due to the low temperatures maintained throughout the freeze-drying process, which facilitated the retention of the original aroma of PCH. Additionally, V-D samples showed a notable sweet sensory profile. Polysaccharides represent 4–10% of PCH’s compositional matrix, with thermal processing inducing partial dehydration-driven caramelization that releases mildly sweet volatiles [50]. The detectable sweetness in the aromatic profile was primarily derived from cedrol (tricyclic sesquiterpene alcohol), whereas differentiation in vegetative olfactory (green) characteristics was governed by lipid-derived C7/C9 aldehydes (heptanal/nonanal) via Maillard-adjacent pathways.

**Figure 7:**
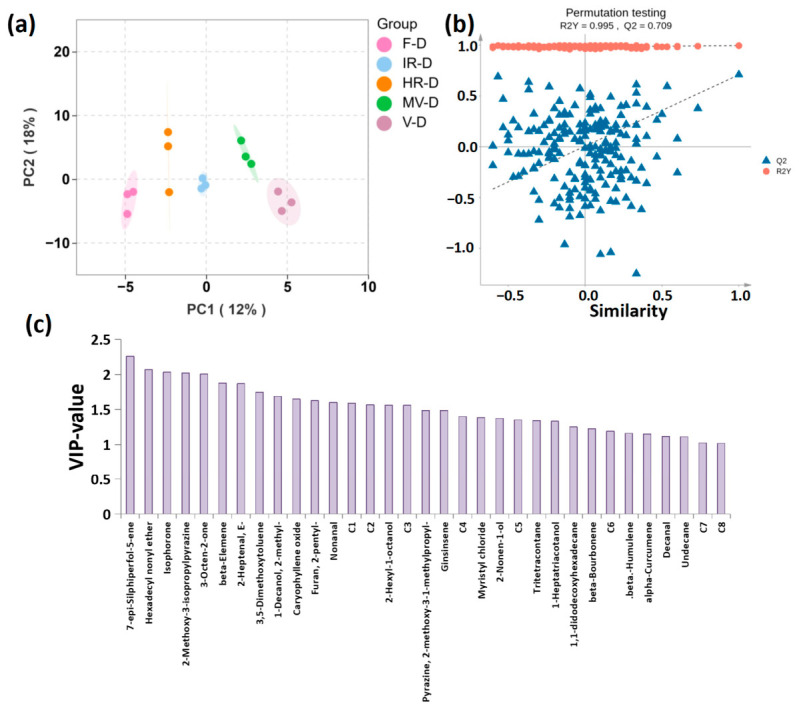
OPLS-DA classification map (**a**), 200 permutation test plots of the OPLS-DA model (**b**); The dashed lines indicate trends, VIP value chart of flavor compounds (**c**); C1, 5-Amino-1-methyl-1H-pyrazole-4-carboxamide, 3TMS; C2, 4,5′-Dibenzamido-1,1′-iminodianthraquinone; C3, 1,4,4a,5,6,7,8,8a-octahydro-2,5,5,8a-tetramethyl-1-Naphthalenemethanol; C4, 1-1-methyl-3-1-methylethyl-1H-pyrazol-5-yl-Ethanone; C5, 9′difluorene-2,2′-dicarboxylic acid, 7,7′-dinitro-Spiro9; C6, 4-methylene-1-methyl-2-2-methyl-1-propen-1-yl-1-vinyl-Cycloheptane; C7, L-Fucose, tetrakistrifluoroacetate, benzyloxime isomer 2; C8, N′-[3,5-Dibromo-2-(2-furoyloxy)benzylidene]-2-(p-tolyloxy)acethydrazide.

The flavor compounds were further categorized based on their functional groups and systematically compared and analyzed from the perspective of chemical properties. Chromatographic profiling revealed nine characteristic volatile groups in dried PCH, comprising alcohols, alkenes, alkanes, esters, acids, ketones, ethers, aldehydes, and others (Figure 6c). Among these, the F-D and IR-D profiles were predominantly composed of aldehydes, the V-D profile mainly constituted aldehydes and acids, and the H-D profile was primarily characterized by alkanes. Given that the entire process is conducted within a low-temperature vacuum environment, the F-D method was capable of preserving the original flavor of fresh PCH to the greatest extent. This, in turn, serves as a valuable reference for maintaining the fresh flavor of PCH, as evidenced by Bhatta et al. [51]. Conducting a thorough comparative analysis revealed that V-D and F-D samples exhibited the most similar composition ratios of flavor compounds, with minimal differences in the proportions of each compound between the two methods. This indicated that the V-D method largely retained the flavor characteristics of fresh PCH. Furthermore, an in-depth analysis and comparison were conducted on the top 20 high-content compounds (Figure 6d). Hexanal was identified as the highest-content compound in F-D, IR-D, and V-D samples, followed by hexanoic acid. By contrast, the highest-content compound in M-D samples was 2,6-dimethylpyrazine, followed by 2-ethyl-3-methylpyrazine. Additionally, a partial least squares discriminant analysis (OPLS-DA) model was constructed by integrating all flavor peak data to further elucidate the differential characteristics. As depicted in Figure 7b, the R^2^Y value was 0.995, and the Q2 value was 0.709. All five drying techniques could be clearly distinguished from one another. In the results of 200 mutagenesis experiments, the regression line exhibited a consistently linear upward trend, with the intercept between Q2 and the *Y*-axis being negative (Figure 7b). This indicated that the model possessed high reliability and strong explanatory power [52]. Further analysis of the variable importance in projection (VIP) values revealed that 33 odor ingredients, including compounds such as isophorone, nonanal, and undecane, exhibited VIP values exceeding 1.0 (Figure 7c). These results indicate that these compounds may function as critical markers for differentiating the five drying methods and elucidating the variations in PCH smell.

In general, with respect to flavor characteristics, M-D and IR-D samples demonstrated more complex flavor profiles and higher concentrations. This is mainly due to the considerable localized thermal impacts that arise during drying. These impacts trigger changes in inherent components, such as the conversion of PCH polysaccharides into caramel, thereby leading to the formation of a diverse range of flavor compounds. According to the 2020 edition of the Chinese Pharmacopoeia, the aroma of dried PCH is characterized as “slightly sweet,” with a polysaccharide content of no less than 7.0% serving as the quality standard. This suggests that the caramel flavor and other flavors generated during the drying process are consistent with the final quality requirements for PCH dried products. By comparison, V-D samples exhibited a flavor intensity and compound class proportions that were more closely aligned with those of F-D samples. Both methods effectively preserved the original flavor profiles of PCH, thereby more fully complying with the odor standards for dried PCH products.

## 4. Conclusions

In this study, a systematic comparison and analysis were conducted on various drying methods for PCH. The results indicate that the Midilli model can effectively fit the process curve of PCH under diverse drying conditions. Regarding phenotypic characteristics, V-D is more effective in preserving the original morphology of PCH. With respect to active constituents, significant variations were observed in total flavonoids, total polyphenols, total polysaccharides, soluble sugars, and antioxidant activity in PCH following different drying methods. Specifically, M-D achieves the highest retention rates of total flavonoids and total polyphenols, whereas V-D demonstrates superior preservation of total polysaccharides and soluble sugars. Moreover, M-D yields the strongest antioxidant activity. Overall, H-D, as a well-established drying technology, exhibits relatively balanced performance across multiple performance indicators but lacks significant advantages over other methods. Although IR-D achieves a faster drying rate and lower energy consumption, it performs less satisfactorily in terms of retaining the color, microstructure, active component content, and oxidation resistance of PCH. On the other hand, M-D not only accomplishes rapid drying and low energy usage but also yields strong antioxidant properties and excellent preservation of total polyphenols and total flavonoids. However, its retention rate of total polysaccharides is relatively low, failing to meet the polysaccharide content requirements specified in the Pharmacopoeia of the People’s Republic of China (2020 edition). On the other hand, although V-D does not exhibit superior drying characteristics, it effectively preserves the cellular structure, color properties, and BI of PCH. Furthermore, V-D performs exceptionally well in retaining the polysaccharide and soluble sugar contents of PCH. Additionally, the flavor characteristics of V-D samples are highly comparable to those of F-D samples.

This study still has certain limitations that need to be improved and refined in future research. For instance, in terms of quality characterization, the evaluation factors selected in this study were relatively limited and failed to cover all quality characteristics. Therefore, in subsequent experiments, we will improve the scope of evaluation factors and focus on quality characteristics. Additionally, based on the research results at the laboratory scale, we will conduct pilot-scale research, using industrial-scale equipment to optimize and verify the production process parameters, in order to ensure that the laboratory research results can be smoothly transferred to the practical production of PCH slices. This will significantly improve product quality and provide a scientific basis for the industrial-scale production of PCH. Furthermore, it is suggested that future research explore emerging new and hybrid drying technologies in order to select the optimal drying scheme that can not only improve drying efficiency but also retain the original quality of PCH slices to the greatest extent.

## Figures and Tables

**Table 1:** Information of thin-layer drying mathematical models.

No.	Model Name	Model Equation	Parameters
1	Lewis	MR = exp(−kt)	k
2	Henderson and Pabis	MR = a exp(−kt)	a, k
3	Parabolic	MR = a + bt + ct^2^	a, b, c
4	Wang and Singh	MR = 1 + at + bt^2^	a, b
5	Midilli	MR = a exp(−kt^n^) + bt	a, k, n, b
6	Two-term exponential	MR = a exp(−kt) + (1 − a)exp(1 − kat)	a, k

**Table 2:** Moisture effective diffusion coefficients of PCH drying.

Sample	Formula for Fitting Linear Regression (lnMR vs. Time (min))	*R* ^2^	Deff/m^2^·s^−1^
H-D	LnMR = −0.019t + 0.574	0.947	2.297 × 10^−10^
M-D	LnMR = −0.144t + 0.151	0.943	1.741 × 10^−9^
IR-D	LnMR = −0.044t + 0.156	0.996	5.320 × 10^−10^
V-D	LnMR = −0.004t + 0.116	0.986	4.836 × 10^−11^
F-D	LnMR = −0.005t + 0.182	0.939	7.255 × 10^−11^

**Table 3:** Mathematical model of drying dynamics and fitting results.

Model Name	Drying Types	Model Parameters	R^2^	χ^2^	RMSE
Lewis	H-D	k = 0.0059	0.94468	0.003292	0.057372
	M-D	k = 0.0746	0.96923	0.0014	0.037417
	IR-D	k = 0.02242	0.98108	0.001294	0.035978
	V-D	k = 0.00215	0.98564	0.00061	0.024707
	F-D	k = 0.00186	0.98702	0.000892	0.029866
Henderson and Pabis	H-D	k = 0.00558, a = 0.94452	0.9472	0.00288	0.053666
	M-D	k = 0.07928, a = 1.038	0.97337	0.001119	0.033456
	IR-D	k = 0.02263, a = 1.00775	0.98003	0.001286	0.035862
	V-D	k = 0.00199, a = 0.94993	0.99451	0.000223	0.01492
	F-D	k = 0.00182, a = 0.97931	0.98649	0.000825	0.028723
Parabolic	H-D	a = 0.96756, b = −0.00546, c = 9.80711 × 10^−6^	0.98907	0.000542	0.023288
	M-D	a = 0.99176, b = −0.05157, c = −9.9224 × 10^−9^	0.99744	9.86 × 10^−5^	0.009928
	IR-D	a = 0.96988, b = −0.01734, c = 8.94665 × 10^5^	0.97073	0.001768	0.042045
	V-D	a = 0.95521, b = −0.00186, c = 1.29058 × 10^−6^	0.99646	0.000137	0.011703
	F-D	a = 0.98326, b = −0.00166, c = 8.80295 × 10^−7^	0.99653	0.000185	0.013601
Wang and Singh	H-D	a = −0.00581, b = 1.05739 × 10^−5^	0.98719	0.000758	0.275717
	M-D	a = −0.05401, b = 5.10848 × 10^−5^	0.99742	0.000117	0.266943
	IR-D	a = −0.01872, b = 1.02562 × 10^−4^	0.97062	0.001892	0.239159
	V-D	a = −0.00213, b = 1.618 × 10^−6^	0.99012	0.000419	0.207529
	F-D	a = −0.00172, b = 9.23025 × 10^−7^	0.99622	0.000257	0.315696
Midilli	H-D	a = 0.99854, b = 0.000579768, n = 1.1216, k = 0.00478	0.99951	2.17 × 10^−5^	0.004663
	M-D	a = 0.99538, b = −0.05241, n = 9.44024 × 10^−9^, k = 0.00462	0.99715	0.0001	0.01
	IR-D	a = 1.02038, b = −0.00111, n = 0.86511, k = 0.03262	0.97894	0.001187	0.034456
	V-D	a = 0.98595, b = 1.04409 × 10^−5^, n = 0.88163, k = 0.0043	0.99771	8.43 × 10^−5^	0.009184
	F-D	a = 0.99872, b = 0.000115714, n = 1.08637, k = 0.00136	0.99683	0.000145	0.012042
Two-term exponential	H-D	a = 1.02322, k = 0.00558	0.96688	0.001806	0.042499
	M-D	a = 0.9848, k = 0.0817	0.96915	0.001296	0.035996
	IR-D	a = 0.99982, k = 0.02245	0.9799	0.001294	0.035978
	V-D	a = 1.02209, k = 0.00211	0.99326	0.000273	0.016537
	F-D	a = 1.00798, k = 0.0018	0.98958	0.000636	0.025219

## Data Availability

The original contributions presented in this study are included in the article/Supplementary Materials. Further inquiries can be directed to the author.

## Supplementary Materials

The following supporting information can be downloaded at: https://www.mdpi.com/article/10.3390/foods15112035/s1, Figure S1: The total ion current diagrams of the PCH obtained by five drying methods; Table S1: Regression Equations and Their Linear Ranges; Table S2: Moisture effective diffusion coefficients of Polygonatum cyrtonema Hua drying; Table S3: Mathematical model of drying dynamics and fitting results.
