# Supplementary material for: Evaluation of Various Drying Methods for Polygonatum cyrtonema Hua: Effects on Drying Characteristics and Multidimensional Quality Assessment
Source: Foods. 2026 Jun 5;15(11):2035. doi: 10.3390/foods15112035 (PMC13257160; doi:10.3390/foods15112035)
Supplement: Supplementary file 1 [file foods-15-02035-s001.zip › foods-4344655-supplementary.pdf]

Supporting information for

Evaluation of Various Drying Methods for *Polygonatum cyrtonema* Hua: Effects on Drying Characteristics and Multidimensional Quality Assessment

Yanbin Wang and Liling Wang*

The Key Laboratory of Biochemical Utilization of Zhejiang Province, Zhejiang Academy of Forestry, Hangzhou 310023, China

* Correspondence: echo22239@163.com

1. Reagents and materials

Polygonatum cyrtonema Hua (PCH) was collected from Liangxi Village, Zhuyang She Township, Lishui City, Zhejiang Province, China (118°56'47.92"E, 28°03'5.99"N, altitude 331.1 m). The samples were authenticated by Dr. Liu Bentong, a senior researcher at the Zhejiang Academy of Forestry Science. The samples were subsequently stored in cold storage at 4°C until used. Prior to the formal experiments, the average moisture content of the fresh extract was determined to be 80.25% (wet base, w.b.) and 432.17% (dry base, d.b.) under room temperature conditions. Subsequently, a multi-functional slicer (model DYQ-401B, manufactured by Rui'an Yongli Pharmaceutical Machinery Co., Ltd., Zhejiang Province, China) was employed to slice the PCH into irregular circular sheets with a thickness of 2 ± 0.2 mm. The device's cutting range was 0.3–3 mm, with an error tolerance of ±10% for subsequent experimental procedures. At this stage, the average initial water content of the sample was measured at 86.87% (w.b.) and 584.18% (d.b.).

High-performance liquid chromatography (HPLC) was employed to quantify the contents of sucrose, glucose, trehalose, fructose, and other components. The experimental reagents included chromatography-grade acetonitrile, phosphoric acid, and methanol. Additionally, Folin-Ciocalteu reagent, gallic acid, sodium nitrite, potassium persulfate, anhydrous sodium carbonate, rutin, sodium hydroxide, non-hydrated aluminum nitrate, ABTS

[(2,2'-azino-bis(3-ethylbenzothiazoline-6-sulfonic acid)] radical cation, and DPPH (1,1-diphenyl-2-picrylhydrazyl) were utilized for the determination of total phenolic content, total flavonoid content, and antioxidant activity. Sulfuric acid and phenol were also employed for the quantification of total polysaccharide content.

2. Drying methods

In each replicate experiment, PCH slice samples weighing 100 ± 0.5 g were randomly selected and subjected to drying treatment. All samples were uniformly distributed on a single carrier tray fabricated from 304 stainless steel. The tray featured a straight mesh structure with a 30-mesh (0.6 mm) aperture and dimensions of 640 × 460 × 45 mm. This design was intended to minimize the contact area between the samples and the tray, thereby reducing heat transfer losses and enhancing drying efficiency. All drying experiments were performed in a temperature-controlled laboratory environment. In accordance with the provisions of the Chinese Pharmacopoeia (2020 Edition) and drying test experience, the drying process was terminated when the moisture content of the PCH samples reached the safety standard of 15% (wet-basis, w.b.).

Microwave Drying (M-D): The sample was positioned in a microwave drying oven (model PM20A1, manufacturer: Midea Group Co., Ltd., Jiangsu, China) for the drying process. The microwave operational frequency was set at 2450 MHz, with a rated input power of 3200 W. The ambient relative humidity was maintained at 4.00 ± 0.50%. During the experiment, the sample was removed at 1-minute intervals, and its mass was measured to document the mass variation during each drying interval.

Infrared drying (IR-D): In the infrared drying process, the sample to be dried is positioned on the tray of an infrared drying oven (Model WS70-1, manufactured by Hangzhou Qiwei Instrument Co., Ltd.), maintaining a distance of 10 cm from the infrared heat source. The equipment is equipped with two infrared bulbs, each rated at 275 W, with an insulation resistance of 10 MΩ, total power output of 550 W, and ambient relative humidity controlled at 4.00 ± 0.50%. During the experiment, the sample mass was recorded every 5 min to monitor the mass changes during the drying process.

Hot air drying (H-D): The sample to be dried is evenly distributed in a hot air drying oven (Model

GZX-9076 MBE, manufactured by Shanghai Boxun Industrial Co., Ltd., Medical Equipment Factory, Shanghai, China). The instrument parameters are set as follows: temperature at 50 °C, wind speed at 0.5 $m \cdot s^{-1}$, relative humidity at 6.00 ± 0.50%, and working power at 1600 W. During the experiment, samples were removed and weighed every 30 min to document the mass changes during the drying process.

Vacuum drying (V-D): The sample to be dried is evenly placed in a vacuum oven (Model DZF-6020, manufactured by Shanghai Yiheng Scientific Instrument Co., Ltd., Shanghai, China), followed by activation of the vacuum pump. When the relative pressure reaches -0.085 MPa, the vacuum process is halted. The drying temperature is set to 50 °C, and the instrument operates at a power of 1400 W. During the experiment, the sample mass was measured every 30 min to record the mass changes during the drying process.

Freeze-drying (F-D): The sample to be dried was first subjected to pre-freezing at -80 °C for a duration of 2 hours. Subsequently, it was transferred to a vacuum freeze-drying oven (Model SCIENTZ-12, manufactured by Ningbo Xinzhi Biotechnology Co., Ltd., Ningbo, China) for the drying process. The vacuum pressure was maintained at 6.0 Pa, the cold trap temperature was set to -50 °C, and the instrument's power rating was 1100 W. During the experiment, the sample mass was recorded at intervals of 120 minutes to monitor the quality changes throughout the drying process.

Table S1. Regression Equations and Their Linear Ranges.

	Linear equation	R^2
Fructose	y=1.558x-0.271	0.997
Glucose	Y=1.504x-0.237	0.999
Sucrose	Y=1.597x-0.337	0.997
Trehalose	Y=1.170x+1.025	0.991

Table S2. Moisture effective diffusion coefficients of *Polygonatum cyrtonema* Hua drying.

Sample	The formula for fitting linear regression (lnMR-Time (min))	R^2	Deff/$m^2 \cdot s^{-1}$
H-D	LnMR=-0.019t+0.574	0.947	2.297×10^{-10}
M-D	LnMR=-0.144t+0.151	0.943	1.741×10^{-9}
IR-D	LnMR=-0.044t+0.156	0.996	5.320×10^{-10}
V-D	LnMR=-0.004t+0.116	0.986	4.836×10^{-11}
F-D	LnMR=-0.005t+0.182	0.939	7.255×10^{-11}

Table S3. Mathematical model of drying dynamics and fitting results.

Model name	Drying types	Model Parameters	R^2	X^2	RMSE
Lewis	H-D	k=0.0059	0.94468	0.003292	0.057372
	M-D	k=0.0746	0.96923	0.0014	0.037417
	IR-D	k=0.02242	0.98108	0.001294	0.035978
	V-D	k=0.00215	0.98564	0.00061	0.024707
	F-D	k=0.00186	0.98702	0.000892	0.029866
Henderson and Pabis	H-D	k=0.00558,a=0.94452	0.9472	0.00288	0.053666
	M-D	k=0.07928,a=1.038	0.97337	0.001119	0.033456
	IR-D	k=0.02263,a=1.00775	0.98003	0.001286	0.035862
	V-D	k=0.00199,a=0.94993	0.99451	0.000223	0.01492
	F-D	k=0.00182,a=0.97931	0.98649	0.000825	0.028723
Parabolic	H-D	a=0.96756,b=-0.00546, c=9.80711E-06	0.98907	0.000542	0.023288
	M-D	a=0.99176,b=-0.05157, c=-9.9224E-09	0.99744	9.86E-05	0.009928
	IR-D	a=0.96988,b=-0.01734, c=8.94665E05	0.97073	0.001768	0.042045
	V-D	a=0.95521,b=-0.00186, c=1.29058E-06	0.99646	0.000137	0.011703
	F-D	a=0.98326,b=-0.00166, c=8.80295E-07	0.99653	0.000185	0.013601
Wang and Singh	H-D	a=-0.00581,b=1.05739E-05	0.98719	0.000758	0.275717
	M-D	a=-0.05401,b=5.10848E-05	0.99742	0.000117	0.266943
	IR-D	a=-0.01872,b=1.02562E-04	0.97062	0.001892	0.239159
	V-D	a=-0.00213, b=1.618E-06	0.99012	0.000419	0.207529
	F-D	a=-0.00172, b=9.23025E-07	0.99622	0.000257	0.315696
Mdilli	H-D	a=0.99854, b=0.000579768, n=1.1216, k=0.00478	0.99951	2.17E-05	0.004663
	M-D	a=0.99538, b=-0.05241, n=9.44024E-09, k=0.00462	0.99715	0.0001	0.01
	IR-D	a=1.02038, b=-0.00111, n=0.86511, k=0.03262	0.97894	0.001187	0.034456
	V-D	a=0.98595, b=1.04409E-05, n=0.88163, k=0.0043	0.99771	8.43E-05	0.009184
	F-D	a=0.99872, b=0.000115714, n=1.08637, k=0.00136	0.99683	0.000145	0.012042
Two-term exponential	H-D	a=1.02322, k=0.00558	0.96688	0.001806	0.042499
	M-D	a=0.9848, k=0.0817	0.96915	0.001296	0.035996
	IR-D	a=0.99982, k=0.02245	0.9799	0.001294	0.035978
	V-D	a=1.02209, k=0.00211	0.99326	0.000273	0.016537
	F-D	a=1.00798, k=0.0018	0.98958	0.000636	0.025219

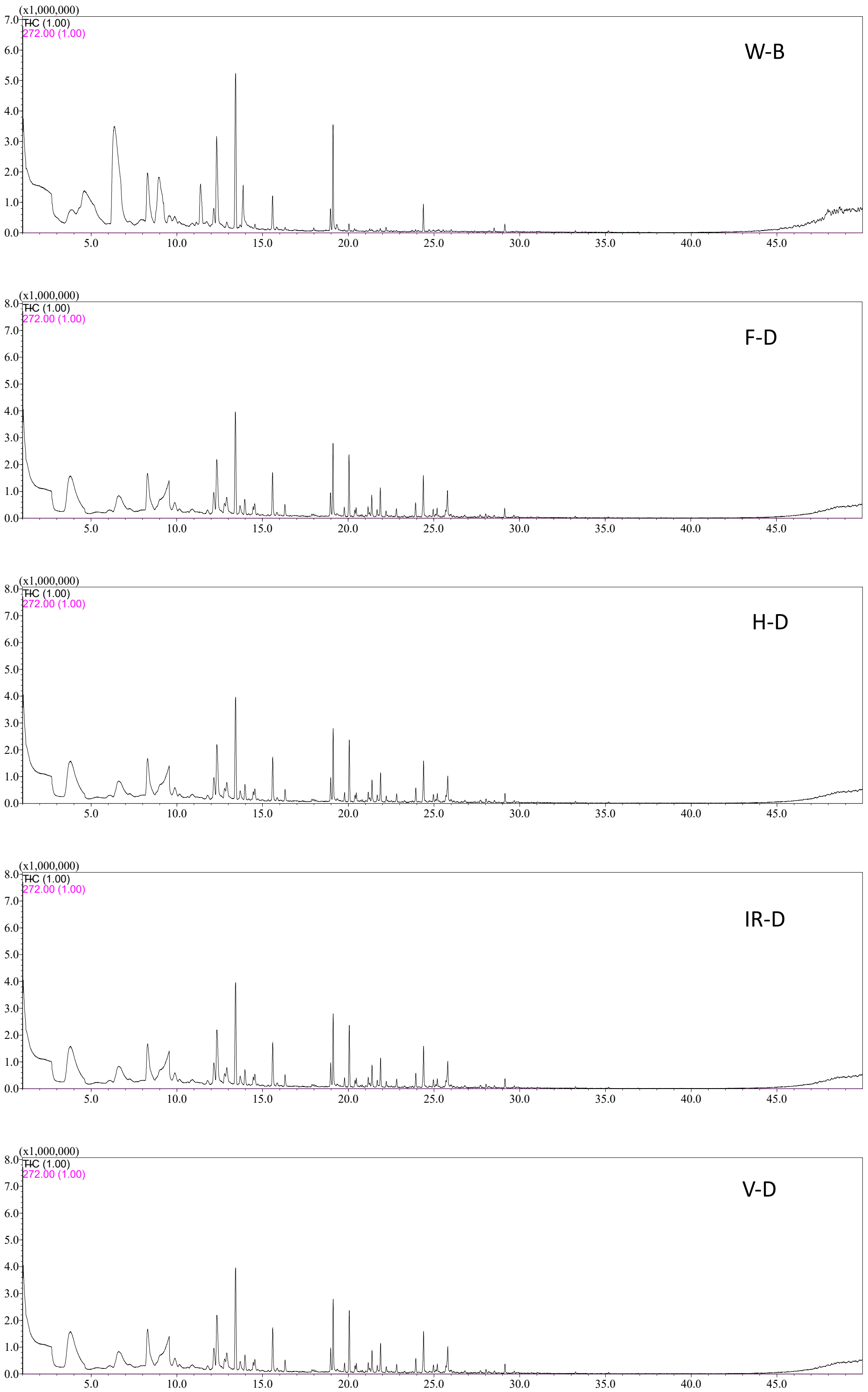

Figure S1. The total ion current diagrams of the PCH obtained by five drying methods.
